# Achievements and futures of immune checkpoint inhibitors in non-small cell lung cancer

**DOI:** 10.1186/s40164-019-0143-z

**Published:** 2019-08-22

**Authors:** Zhenbin Qiu, Zihao Chen, Chao Zhang, Wenzhao Zhong

**Affiliations:** Guangdong Lung Cancer Institute, Guangdong Provincial Key Laboratory of Translational Medicine in Lung Cancer, Guangdong Provincial People’s Hospital, Guangdong Academy of Medical Sciences, No. 106, Zhongshan Er Road, Yuexiu District, Guangzhou, 510080 Guangdong China

**Keywords:** Checkpoint inhibitors, NSCLC, Novel therapies

## Abstract

Non-small cell lung cancer (NSCLC) has been threatening human health for years. Cytotoxicity-based chemotherapy seems to reach plateau in NSCLC treatment. Immunotherapy with immune checkpoint inhibitors (ICIs) against programmed cell death 1 (PD-1/L1) axis are to provide long-term survival benefits for wild-type advanced NSCLC patients with acceptable adverse effects. Though beneficiary population is limited from monotherapy, combination strategies are expanding indicators. Retrospective evidences suggested ICIs are also potentially useful for brain metastasis. Furthermore, the combination of ICIs and surgery are to prolong progression free survival time for local advanced patients. Additionally, novel agents targeting in immune checkpoints other than PD-1/L1 demonstrated potential values in anticancer immunity. Herein, we summarize the novel therapies of checkpoint inhibitors in NSCLC treatment and some other potential immunotherapy to provide a conspectus for novel immunotherapy in NSCLC and perspective for the future in anti-cancer treatment.

## Background

Lung cancer is the leading cause of cancer related death worldwide, including about 85% non-small cell lung cancer (NSCLC) [1]. Platinum-based chemotherapy had long been standard treatment for advanced NSCLC, with only about 7.9 months median overall survival (OS) [2]. Target therapy has already reformed the treatment of NSCLC harboring driver oncogene mutation, which significantly prolong the survival time [3]. Meanwhile, the progression of immunotherapy in recent years has also greatly promoted the treatment of driving gene negative NSCLC, with longer survival and miner adverse reaction than chemotherapy [4].

To our knowledge, through eliminating mutated cell, cytotoxic T lymphocyte (CTL) can prevent cancer’s development and progression [5]. CTL’s function is regulated by complicated immune signal pathways [6]. In cancer immunity, T cell receptor (TCR) recognizes major histocompatibility complex (MHC) with cancer-specific antigen as first signal to activate CTLs. And the second signal is from costimulatory factors, also called immune checkpoints, including stimulators for maintaining activation and inhibitors for preventing over-activation. For example, programed cell death-1 (PD-1) is such kind of inhibitor, which expressed in activated CTLs. Once PD-1 binds to its ligand, programed cell death ligand 1 (PD-L1) highly expressed in tumor cell’s (TC) membrane, CTL’s recognition to TCs would be suppressed, so that TCs could achieve self-adaption and escape from immune elimination [7]. Accordingly, immune checkpoint inhibitors (ICIs) can block that kind of pathways and rebuild the CTL’s ability to clear malignant cells.

Efficacy of ICIs, targeting in PD-1/PD-L1 axis, in NSCLC’s clinical treatment has been proved a lot, from second line to first line, even in early stage patients [8–12]. Long-term follow-up data demonstrated immunotherapy has great potential for long-term response [13]. However, limited beneficiary population and drug resistance are hindering the further development of immunotherapy [14]. With deeper research on anticancer immunity, novel immune checkpoint inhibitors are desired to expand beneficiary population from immunotherapy [15–18]. Herein, we summarize the novel therapies of checkpoint inhibitors in NSCLC treatment and some other potential immunotherapy to provide a conspectus for novel immunotherapy in NSCLC and perspective for the future in anti-cancer treatment.

## Immune checkpoint inhibitors in advanced NSCLC

### Pembrolizumab, nivolumab and atezolizumab

Immune checkpoint inhibitors have made a great progression in advanced NSCLC without positive driver mutation in recent years [8–12]. The efficacy of single agent therapy with ICIs is proved from second line to first line. Due to the efficacy and safety demonstrated by pembrolizumab in keynote 001 [9], FDA approved it as an optional second-line treatment for advanced NSCLC. Subsequently, keynote 010 further proved that compared with docetaxel, PD-L1 positive (tumor proportion score, TPS ≥ 1%) patients could gain significantly survival benefits from pembrolizumab, especially those with TPS ≥ 50%, and the incidence of adverse reactions was lower [10]. Moreover, results of keynote 024 and 042 supports the use of pembrolizumab monotherapy as first line treatment for PD-L1 positive NSCLC patients, especially for high expression population [19, 20]. Despite lacking evidence of benefits from first line treatment, patients can also obtain survival and life quality benefits from monotherapy with nivolumab or atezolizumab after failing in first line treatment because of the positive results of checkmate 017/057 and OAK [11, 12] (Table 1).

**Table 1:** Posted results of monotherapy with pembrolizumab, atezolizumab, nivolumab, durvalumab and avelumab in advanced NSCLC

Identifier	Trials	Agent	phase	Indication	Population	Arms	Biomarkers	ORR	mPFS	mOS	mDOR	Adverse effects (grade ≥ 3)
NCT01295827	Keynote 001	Pembrolizumab	I	Second line	Advanced NSCLC	Pembrolizumab	Regardless PD-L1	19.40%	3.7 m	12.0 m	NR	9.50%
							PD-L1 ≥ 50%	45.20%	6.3 m	NR	12.5 m	NA
NCT01905657	Keynote 010	Pembrolizumab	II/III	Second line	Previously treated non-small-cell lung cancer with PD-L1 expression on at least 1% of tumour cells	Pembrolizumab = 2 mg/kg	PD-L1 ≥ 50%	30.00%	5.0 m	14.9 m	NR	13.00%
						Pembrolizumab = 10 mg/kg	PD-L1 ≥ 50%	29.00%	5.2 m	17.3 m	NR	16.00%
						Docetaxel	PD-L1 ≥ 50%	8.00%	4.1 m	8.9 m	8 m	35.00%
NCT02142738	Keynote 024	Pembrolizumab	III	First line	Previously untreated advanced NSCLC with PD-L1 expression on at least 50% of tumor cells and no mutation of EGFR or ALK	Pembrolizumab 200 mg/3 weeks	PD-L1 ≥ 50%	44.80%	10.3 m	NR	NR	26.60%
						Platinum-based chemotherapy		27.80%	6.0 m	14.5 m	6.3 m	53.30%
NCT02220894	Keynote 042	Pembrolizumab	III	First line	Previously untreated advanced non-small-cell lung cancer without a sensitising EGFR mutation or ALK translocation and with ECOG 0 or 1, and a PD-L1 TPS of 1% or greater	Pembrolizumab	PD-L1 ≥ 50%	39.50%	7.1 m	20.0 m	20.2 m	17.80%
							PD-L1 ≥ 20%	33.40%	6.2 m	17.7 m		
							PD-L1 ≥ 1%	27.30%	5.4 m	16.7 m		
						Platinum-based chemotherapy	PD-L1 ≥ 50%	32.00%	6.4 m	12.1 m	10.8 m	41.90%
							PD-L1 ≥ 20%	28.90%	6.6 m	13.0 m	8.3 m	
							PD-L1 ≥ 1%	26.50%	6.5 m	12.1 m	8.3 m	
NCT02008227	OAK	Atezolizumab	III	Second line	Previously treated NSCLC	Atezolizumab	ITT population	14.00%	2.8 m	13.8 m	16.3 m	15.00%
						Docetaxel		13.00%	4.0 m	9.6 m	6.2 m	43.00%
NCT01642004/NCT01673867	Checkmate 017/057	Nivolumab	III	Second line	Previously treated patients with advanced squamous or nonsquamous non–small-cell lung cancer	Nivolumab	NA	NA	2.56 m	11.1 m	Squamous: 25.2 m	10.00%
											Non-squamous: 17.2 m	
						Docetaxel		NA	3.52 m	8.1 m	Squamous: 8.4 m	55.00%
											Non-squamous: 5.6 m	
NCT01693562	Study 1108	Durvalumab	I/II	Second line	Pretreated NSCLC EGFR/ALK wild type	Durvalumab from 0.1 to 10 mg/kg q2w or 15 mg/kg q3w	PD-L1 ≥ 25%	25.30%	2.8 m	15.4 m	NR	10.00%
							PD-L1<25%	6.10%	1.5 m	7.6 m		
				First line	Treatmentnaïve advanced NSCLC EGFR/ALK wild type	Durvalumab 10 mg/kg q2w	PD-L1 ≥ 25%	28.60%	4.0 m	21 m	NR	9.00%
							PD-L1<25%	11.00%	NA	NA		
NCT02220894	ATLANTIC	Durvalumab	II	Third line	Heavily pretreated advanced NSCLC EGFR/ALK positive	Durvalumab	PD-L1 ≥ 25%	14.10%	1.9 m	13.3 m	7.4 m	5.40%
							PD-L1<25%	3.60%	1.9 m	9.9 m	NR	
					Heavily pretreated advanced NSCLC EGFR/ALK wild type or unknown	Durvalumab	PD-L1 ≥ 90%	30.90%	2.4 m	NR	NR	17.60%
							PD-L1 ≥ 25%	7.50%	3.3 m	10.9 m		8.20%
							PD-L1<25%	3.30%	1.9 m	9.3 m		
NCT02766335	Lung-Map	Durvalumab	II	Second line	Pretreated NSCLC EGFR/ALK wild type	Durvalumab	PD-L1 ≥ 25%	14.30%	NA	10.7 m	NR	34.00%
							PD-L1<25%	6.90%	NA	11.6 m		
						Docetaxel	NA	6.70%	NA	7.7 m	NR	NA
NCT02125461	PACIFIC	Durvalumab	III	Second line	Unresectable stage III NSCLC after chemoradiation Regardless of PD-L1 status	Durvalumab	NA	28.40%	16.8 m	23.2 m	NR	29.90%
						Placebo		16.00%	5.6 m	14.6 m		26.10%
NCT02395172	JAVELIN Lung 200	Avelumab	III	Second line	Platinum-treated patients with advanced NSCLC	Avelumab	PD-L1 ≥ 1%	19.00%	3.4 m	11.4 m	NR	10.00%

Patients can obtain survival and life quality benefits from monotherapy with tolerable adverse reactions

*ORR* objective response rate, *mPFS* median Progression Free Survival, *mOS* median Overall survival, *mDOR* median Duration of Response, *NR* not reached, *NA* nona, *ITT* intend to treat

Though single agent therapy with ICIs has already reformed the treatment strategy of advanced NSCLC, there is still a great proportion of patients could not respond [21]. Combination strategies may help to overcome the resistance (Table 2).

**Table 2:** Posted results of first-line combination regimen trials for pembrolizumab, nivolumab, and atezolizumab in advanced NSCLC

Identifier	Trials	Agent	Phase	Population	Arms	Biomarkers	ORR	mPFS	1 year PFS rate	mOS	1 year OS rate	mDOR	Adverse Effects Rate (≥ grade 3)
NCT02039674	Keynote 021	Pembrolizumab	II	Untreated metastatic Non-squamous NSCLC Without EGFR/ALK alteration	Pembrolizumab + platinum	Regardless PD-L1	55.00%	24 m	NA	NR	NA	NA	39.00%
					Platinum		29.00%	9.3 m		21.1 m			26.00%
NCT02578680	Keynote 189	Pembrolizumab	III	Untreated metastatic Non-squamous NSCLC Without EGFR/ALK alteration	Pembrolizumab + platinum	Regardless PD-L1	47.60%	8.8 m	34.10%	NR	69.20%	11.2 m	67.20%
					Platinum		18.90%	4.9 m	17.30%	11.3 m	49.90%	7.8 m	65.80%
NCT02775435	Keynote 407	Pembrolizumab	III	Untreated metastatic, squamous NSCLC	Pembrolizumab + platinum	Regardless PD-L1	57.90%	6.4 m	NA	15.9 m	65.20%	7.7 m	69.80%
					platinum		38.40%	4.8 m	NA	11.3 m	48.30%	4.8 m	68.20%
NCT02477826	Checkmate 227	Nivolumab	III	Untreated metastatic Non-squamous NSCLC Without EGFR/ALK alteration	Nivolumab + platinum	TMB ≥ 10mut/Mb	60.50%	NA	27.00%	NA	NA	NA	NA
					Nivolumab + ipilimumab		45.30%	7.2 m	45.00%				31.20%
					Platinum		27.00%	5.4 m	13.20%				36.10%
NCT02657434	Impower 131	Atezolizumab	III	Treatment-naïve Stage IV squamous NSCLC	Atezolizumoab + carboplatin	ITT population	49.00%	6.3 m	24.00%	14.0 m	55.60%	NA	69.00%
					Carboplatin		41.00%	5.6 m	12.00%	13.9 m	56.80%		58.00%
2NCT02657434	Impower132	Atezolizumab12	III	Non-squamous NSCLC Without EGFR/ALK alterationUntreated metastatic	Atezolizumoab + carboplatin	ITT population	47.00%	7.6 m	33.70%	18.1 m	59.60%	10.1 m	69.00%
					Carboplatin		32.00%	5.2 m		13.6 m			59.00%
NCT02659059	Checkmate 568	Nivolumab	II	Untreated metastatic Non-squamous NSCLC Without EGFR/ALK alteration	Nivolumab plus low-dose ipilimumab	PD-L1 < 1%	41.00%	6.8 m	52%^a^	NA	NA	NA	29.00%
						PD-L1 ≥ 1%	15.00%	2.8 m	32%^a^				
						TMB ≥ 10 mut/Mb	44.00%	7.1 m	55%^a^				
						TMb < 10 mut/Mb	12.00%	2.6 m	31%^a^				

Combination strategies may help patients overcome NSCLC resistance that ICI monotherapy face with

*ORR* objective response rate, *PFS* Progression Free Survival, *OS* overall survival, *DOR* Duration of Response, *ITT* intend to treat, *TMB* tumor mutation burden

^a^6-month PFS rate

It is reported that platinum-based chemotherapy can contribute to sensitization of tumor to ICIs through increasing CD8+ T cell infiltration [22]. Keynote 021 is the first trial which succeed in combining platinum-based chemotherapy and ICIs for treat naïve pan-negative advanced NSCLC [23]. Regardless of PD-L1’s expression, the ORR is almost double in pembrolizumab plus chemotherapy comparing to chemotherapy, while the risks of progression and death are decreasing to only a half with the toxicity safely controlled. After that, keynote 189 and 407 successively announced their similar results in squamous and non-squamous cell carcinoma [24, 25], which have further strengthened evidences for combining ICIs and platinum-based chemotherapy as first line treatment. Comparing with keynote 024/042, for patients with low or negative PD-L1 expression (TPS < 50%), the strategy of combination with chemotherapy is safer and more cost-effective [26]. Additionally, Impower 131 and 132 reached their primary endpoint, proving that patients can gain more survival benefits and less risks from the combination of atezolizumab and chemotherapy rather than monotherapy with chemical agents [27, 28]. Interestingly, in the exploring analysis, both trials are observed that in PD-L1 high expression and negative group, combination presents better PFS than monotherapy, while in PD-L1 low expression group, there is no significant difference between them, indicating the biomarkers for patient selection need to be explored more. After failed in the competition of monotherapy, exploring combination may help nivolumab to break the dilemma in first line treatment. In ASCO 2018 meeting, Borghaei et al. announced a sub-group analysis of checkmate 227, nivolumab plus chemotherapy has a trend in improving PFS comparing to chemotherapy in patients with negative PD-L1 expression (HR = 0.74 [95% CI 0.58, 0.94]) [29]. Furthermore, nivolumab plus chemotherapy can significantly improve 1-year PFS rate (27% vs 8%; HR = 0.56 [95% CI 0.35, 0.91]) in those patients harboring high tumor mutation burden (TMB ≥ 10 Mut/Mb) than chemotherapy, suggesting that high TMB is a good predictor for benefits of combination [29].

Cytotoxic T-lymphocyte association protein 4 (CTLA-4) is another negative immune checkpoint [7]. Differ to PD-1 pathway, CTLA-4 pathway inhibits T cell in the initial stage of activation [30]. Thence, blocking both PD-1 and CTLA-4 pathways could make synergistic effects, which could awake more CTLs in the initial stage of immunity and recover the immune activity in the late stage. Ipilimumab is a human-IgG1 antibody targeting against CTLA-4. The combination of nivolumab and ipilimumab was evaluated in several trials. After efficacy and safety were confirmed in checkmate 012, checkmate 227 was initialed for exploring more evidences [31]. Regardless PD-L1’s status, double ICIs can significantly improve ORR (45.3% vs 26.9%) and median PFS (7.2 m vs 5.4 m) comparing to chemotherapy in high TMB group. It is worth mentioned that combination of two ICIs can achieve higher 1-year PFS (45% vs 27%) than the combination of ICIs and chemotherapy in PD-L1 negative patients with high TMB. Safety is also satisfactory. 31.2% patients in combination group suffered from grade 3/4 AEs, while 36.1% in chemotherapy group [32]. Furthermore, checkmate 568 recently confirmed that PD-L1 positive (TPS ≥ 1%) and high TMB (≥ 10 Mut/Mb) are both independent biomarkers for better effects prediction in such combination as first line treatment [33].

### Durvalumab and avelumab

Durvalumab was first evaluated as a single agent in a large phase 1/2 study in advanced solid tumor patients [34], including refractory advanced NSCLC (NCT01693562). According to prior lines of therapy, the ORR was 27.1% in treatment-naïve vs 18.8% in patients pretreated with platinum-based chemotherapy (second-line). High PD-L1 expression was associated with better response rates (25.3%; 39/154 patients) compared to low PD-L1 expression patients (6.1%;7/115 patients). Antonia et al. [35], reported that according to the line of treatment, the ORR was 26.1% in high PD-L1 vs 4.2% in low PD-L1 in platinum-refractory patients, and 22.0% in high PD-L1 vs 6.1% in low PD-L1 in third or later lines. S1400A Lung-Map umbrella phase 2 trial (NCT02766335) showed durvalumab having an ORR of 14.3% in ≥ 25% PD-L1 expression (n = 14) and 6.9% in low/negative PD-L1 (TPS ≤ 25%) (n = 25) [36]. ATLANTIC phase2 study (NCT02087423) showed PD-L1 expression ≥ 25% with a better median PFS than low/negative PD-L1 population (3.3 m vs 1.9 m) [37]. In a heavily pretreated EGFR/ALK wild-type or unknown population, durvalumab demonstrated activity and durable responses. Trials of durvalumab combination regimens initiated, owing to the great success of durvalumab in second-line treatment as single agent. Avelumab is one of the last PD-L1 inhibitors to access the market, a fully human immunoglobulin G1 (IgG1) monoclonal antibody that specifically binds PDL1 and inhibits its binding to PD-1 [38]. JAVELIN Lung 200 is the first phase 3 trial of avelumab in patients with platinum pretreated NSCLC as monotherapy. Though median overall survival did not differ between avelumab and docetaxel group(11.4 m vs 10.3 m)in full analysis set population (FAS), post hoc analyses identified that specific populations who could benefit from anti-PD-1 or anti-PD-L1 antibodies. In PD-L1 population,median overall survival in subgroup TPS ≥ 80% and ≥ 50% was 17.1 months and 13.6 months in the avelumab group, comparing with 9.2 months in the docetaxel group [39]. These results indicate that most patients with high PD-L1 expression can achieve improved overall survival if given avelumab.

Durvalumab is currently being investigated in combination with different immunotherapies, in the majority of cases with tremelimumab. Durvalumab in combination with tremelimumab was initially assessed in a phase 1/2 study (006, NCT02000947) in 102 treatment-naïve NSCLC patients [40]. Durvalumab demonstrated clinical activity with an ORR of 17% [95% CI 9–29]). Based on the safety and activity, durvalumab 20 mg/kg q4w plus tremelimumab at 1 mg/kg were the recommended dose for phase 3 trials. The phase 3 MYSTIC trial (NCT02453282) enrolled 1092 advanced EGFR/ALK wild-type treatment-naïve NSCLC patients to compare durvalumab plus tremelimumab vs durvalumab vs SoC (platinum-based chemotherapy). In July 2017, MYSTIC trial did not meet the primary endpoint of PFS compared to chemotherapy. The study is continuing as planned to assess the primary endpoint of OS, PFS [41]. Classically, platinum-doublet chemotherapy has been the SoC as first-line therapy for advanced NSCLC, improving survival and quality of life in treatment-naïve patients. However, recently single agent PD-1 inhibitor demonstrated better outcomes compared to platinum-based chemotherapy in ≥ 50% PD-L1 tumors [42], as well as in combination with platinum-based chemotherapy for nonsquamous population, regardless of PD-L1 expression [23, 43]. Based on this potential synergism, several studies are currently evaluating ICI-chemotherapy combinations of durvalumab. For now, the limited evidence available is insufficient to establish the clinical impact of ICIs for EGFR and ALK-positive patients. The population harboring a driver molecular alteration such as an EGFR mutation is generally excluded from the majority of immunotherapy clinical trials. The only data available are derived from subgroup analyses from the phase 3 study with single-agent durvalumab in previously-treated populations, which showed no clear benefit in a small number of EGFR mutated patients [12, 42, 44], and with an insufficient basis to draw definite conclusions. The ALK positive population has also been widely excluded from the majority of immunotherapy clinical trials. Several ongoing studies are evaluating the safety and efficacy of different ICIs as a single agent or in combination with an ALK TKI, however no solid evidence has been reported to date [37]. In the phase 1b TATTON study (NCT02143466) the combination durvalumab plus osimertinib was evaluated in EGFR-mutated patients. However, due to the high incidence of interstitial lung disease (ILD), this study arm was stopped prematurely, as was the phase 3 CAURAL trial (NCT02454933) assessing osimertinib plus durvalumab vs osimertinib in second-line EGFR-mut NSCLC patients. The phase II ATLANTIC trial testing durvalumab as third-line treatment included the largest cohort of EGFRmutant patients treated with ICI (n = 98) after progression on EGFR TKI and chemotherapy. According to PD-L1 expression (< 25% or ≥ 25%), durvalumab achieved a RR of 3.6% and 14.1%, a similar median PFS 1.9 months and a median OS of 9.9 months and 13.3 months, respectively. As the data showed, even patients with heavily pretreated, EGFR/ALK mutation-positive advanced NSCLC may also benefit from greater than or equal to third-line PD-1/PD-L1 inhibitors treatment, with durable efficacy and a promising effect on OS. The most impressive results for immunotherapy and radiation, have come from the phase 3 PACIFIC trial (NCT02125461). It showed positive results with durvalumab significantly reducing the risk of disease worsening or death for stage III unresectable lung cancer. Median PFS was 16.8 months with durvalumab vs 5.6 months with placebo. And ORR was 28.4% with durvalumab vs 16% with placebo (p < 0.001) [45]. The FDA approved durvalumab for the treatment of unresectable stage III NSCLC without progression after treatment with chemotherapy and radiation (chemoradiation) in 2018 [46]. Ongoing phase 3 trials will provide illuminating data to confirm the place of durvalumab in NSCLC patients (Table 3). Both as monotherapy and combination therapy in the JAVELIN Solid Tumor trial [38], avelumab showed a manageable safety profile and promising clinical activity in this population of pretreated metastatic or recurrent NSCLC patients. Regarding its tolerability profile, fatigue (25%) and infusion-related reactions (19%) were the most frequent grade ≥ 3 adverse events. Despite the antitumor activity shown by avelumab in patients with advanced pretreated NSCLC patients, this novel anti-PD-L1 compound still has a long pathway to walk in order to demonstrate its potential clinical utility and own personality, for the first and second line scenario in advanced NSCLC. Ongoing studies will contribute to a better understanding of the efficacy and safety of avelumab (Table 4).

**Table 3:** Ongoing phase III trials for durvalumab in non-small cell lung cancer

Identifier	Title	Interventions	Study design	Population	Primary endpoint	Secondary endpoint	Status	Primary Completion
NCT03800134	A study of neoadjuvant/adjuvant durvalumab for the treatment of patients with resectable stages II and III non-small cell lung cancer (AEGEAN)	Durvalumab + platinum-based chemotherapy Placebo + platinum-based chemotherapy	Randomized parallel trial	Resectable stage IIA–IIIB NSCLC	MPR	pCR, OS, DFS	Recruiting	27-Jul -20
NCT03519971	Study of durvalumab given with chemoradiation therapy in patients with unresectable non-small cell lung cancer (PACIFIC2)	Durvalumab + platinum-based chemotherapy and radiation Placebo + platinum-based chemotherapy and radiation	Randomized parallel trial	Unresectable locally advanced stage III NSCLC	PFS, ORR	OS, DOR, PFS2	Recruiting	30-Sep-20
NCT02273375	Double blind placebo controlled study of adjuvant MEDI4736 in completely resected NSCLC	Durvalumab Placebo	Randomized parallel trial	Stage IB (> 4 cm) to IIIA NSCLC after complete surgical resection	DFS	OS, LCSS	Recruiting	Jan-23
NCT03706690	A study of durvalumab as consolidation therapy in non-small cell lung cancer patients (PACIFIC5)	Durvalumab Placebo	Randomized parallel trial	Unresectable locally advanced stage III NSCLC	PFS	OS, ORR, DOR	Recruiting	25-Mar-21
NCT03164616	Study of durvalumab + tremelimumab with chemotherapy or durvalumab with chemotherapy or chemotherapy alone for patients with lung cancer (POSEIDON)	Durvalumab + tremelimumab Durvalumab monotherapy + SoC SoC chemotherapy alone	Randomized parallel trial	Untreated advanced NSCLC without activating EGFR mutation or ALK fusions	PFS, OS	ORR, DOR,	Recruiting	30-Sep-19
NCT03003962	Study of durvalumab alone or chemotherapy for patients with advanced non small-cell lung cancer	Durvalumab SoC chemotherapy	Randomized parallel trial	Untreated advanced PD-L1 positive NSCLC without EGFR mutation and ALK rearrangement	OS	ORR, DOR, PFS	Active, not recruiting	30-Sep-19
NCT02453282	Phase III open label first line therapy study of MEDI 4736 (durvalumab) with or without tremelimumab versus soc in non-small-cell lung cancer (MYSTIC)	Durvalumab Durvalumab + tremelimumab SoC chemotherapy	Randomized parallel trial	Untreated advanced NSCLC without activating EGFR mutation or ALK fusions	OS, PFS	ORR	Active, not recruiting	4-Oct-18
NCT02542293	Study of 1st Line Therapy Study of Durvalumab With Tremelimumab Versus SoC in Non Small-Cell Lung Cancer (NSCLC) (NEPTUNE)	Durvalumab + tremelimumab SoC chemotherapy	Randomized parallel trial	Untreated advanced NSCLC without activating EGFR mutation or ALK fusions	OS	PFS, ORR, DOR	Active, not recruiting	22-Aug-19

Durvalumab is currently being investigated in combination with different immunotherapies

*DCR* disease control rate, *LCSS* lung cancer-specific survival, *PFS2* time from randomization to second progression, *TTD*/*TTM* time to death/time to distant metastasis, *MPR* major pathological response, *pCR* pathological, complete response, *DFS* disease-free survival

**Table 4:** Ongoing clinical trials for avelumab in non-small cell lung cancer

NCT Number	Title	Phase	Interventions	Study design	Population	Primary endpoint	Secondary endpoint	Status	Primary completion
NCT03050554	Stereotactic body radiation therapy (SBRT) combined with avelumab (anti-PD-L1) for management of early stage non-small cell lung cancer (NSCLC)	I/II	Avelumab + SBRT	Single-arm trial	Stage I NSCLC with tumor(s) less than 5 cm in diameter or 250 cm^3^ in volume	Safety and tolerability, RFS	Locoregional control, OS	Active, not recruiting	01-Oct-20
NCT02576574	Avelumab in first-line non-small cell lung cancer (JAVELIN Lung 100)	III	Avelumab Pemetrexed Paclitaxel Gemcitabine Carboplatin Cisplatin	Randomized control trial	Metastatic or recurrent NSCLC without EGFR or ALK	PFS, OS	BOR, DOR, EQ-5D-5L	Active, not recruiting	07-Jun-20
NCT03717155	Study of avelumab and cetuximab plus gemcitabine and cisplatin in participants with squamous non-small cell lung cancer (NSCLC)	II	Avelumab + cetuximab + gemcitabine + cisplatin	Single-arm trial	Advanced lung squamous carcinoma without EGFR mutation, ALK rearrangementand brain metastasis	Best overall response	Occurrence of treatmentemergent adverse events, PFS, DOR	Recruiting	25-Jan-21
NCT03472560	A study of avelumab in combination with axitinib in non-small cell lung cancer (NSCLC) or urothelial cancer (Javelin Medley VEGF)	II	Avelumab + axitinib	Single-arm trial	Pretreated advanced NSCLC with no more than 2 prior lines and EGFR/ALK/ROS1 negative	ORR	TTR, DOR, PFS	Recruiting	18-Sep-20
NCT02584634	Study to evaluate safety, efficacy, pharmacokinetics and pharmacodynamics of avelumab in combination with either crizotinib or PF-06463922 In patients with NSCLC (Javelin Lung 101)	II	Avelumab Crizotinib	Non-randomized parallel trial	Advanced or metastatic NSCLC. ALK negative or positive	DLTs, ORR	PFS, DOR, TTR	Active, not recruiting	15-Feb-19
NCT03317496	Safety and efficacy study of avelumab plus chemotherapy with or without other anti-cancer immunotherapy agents in patients with advanced malignancies	II	Aveluma + pemetrexed/carboplatin Avelumab + gemcitabine/cisplatin	Non-randomized parallel trial	Untreated advanced non-squamous NSCLC without EGFR mutations or ALK rearrangement	DLT, ORR	PFS, DOR, TTR	Recruiting	04-Sep-20
NCT03268057	VX15/2503 in combination with avelumab in advanced non-small cell lung cancer	I/II	VX15/2503 + avelumab	Single-arm trial	No prior immunotherapy treated NSCLC	DLT, AEs	ORR, DOR, PFS	Recruiting	01-May-20
NCT03270176	A dose-finding study of the second mitochondrial activator of caspases (SMAC) mimetic debio 1143 when given in combination with avelumab to participants with advanced solid malignancies and to participants with advanced or metastatic non-small cell lung cancer (NSCLC) after platinum-based therapy	I	Debio 1143 + avelumab	Single-arm trial	NSCLC of stage IIIB or IV (7th IASLC) that has progressed after one line of platinum containing doublet chemotherapy	Maximum tolerated dose, ORR	SAEs, BOR, DOR, PFS, OS	Recruiting	01-Sep-19
NCT03158883	UCDCC#270: avelumab and stereotactic ablative radiotherapy in non-responding and progressing NSCLC patients	Early I	Avelumab Stereotactic ablative radiotherapy (SAR)	Non-randomized parallel trial	Immunotherapy pretreated advanced NSCLC without EGFR mutations or ALK rearrangement	Overall response rate	OS, PFS, DCR	Recruiting	01-Jun-20
NCT03514719	PD-L1 imaging in non small cell lung cancer’ (PINNACLE)	I	Avelumab	Single-arm trial	Stage IIIb/IV NSCLC or resectable stage Ia (≥ T1b tumor)—IIIa NSCLC	Tumor uptake of 89Zr-Avelumab	Correlation 89Zr-avelumab uptake in tumor lesions and PD-L1 expression	Recruiting	31-Mar-22
NCT03637491	A study of avelumab, binimetinib and talazoparib in patients with locally advanced or metastatic RAS-mutant solid tumors	II	Avelumab Binimetinib Talazoparib	Randomized control trial	Locally advanced (primary or recurrent) or metastatic solid tumors	DLT, ORR	TTR, OS, PFS	Recruiting	01-May-22
NCT03409458	A dose escalation and confirmation study of PT-112 in advanced solid tumors in combination with avelumab	I/II	PT-112 + avelumab	Single-arm trial	Metastatic or locally advanced disease	Recommended dose	DLTs, AEs, ORR, DCR, PFS	Recruiting	01-Feb-20
NCT03386929	Survival prolongation by rationale innovative genomics	I/II	Avelumab + axitinib + palbociclib	Single-arm trial	Locally advanced or metastatic NSCLC	DLT, RR, PFS, OS	Incidence of treatment-related and or biopsy-related serious adverse events	Recruiting	01-Dec-21

Avelumab showed a manageable safety profile and promising clinical activity in pretreated metastatic or recurrent NSCLC patients

*PFS* progression-free survival, *OS* overall survival, *ORR* objective response rate, *DLT* dose-limiting toxicities, *DOR* duration of response, *TTR* time to response, *BOR* best overall response, *TEAE* treatment of adverse events

## Immune checkpoint inhibitor in NSCLC with CNS metastasis

Central nerve system (CNS) metastasis is quite common in advanced NSCLC. And about 40% driver mutation negative patients would suffer from it [47]. Local treatment for CNSs locus has limited efficacy in survival time extension [48]. Systematic immunotherapy has been an important part for advanced NSCLC, but the efficacy in CNSs metastasis patients is still under exploring. A retrospective research conducted in Israel found that nivolumab can provide equal survival benefits for patients with or without CNS metastasis (median OS: 7.0 m vs 5.2, p = 0.5) [49], which means both intracranial and extracranial lesions can benefit from ICIs. For pretreated, stable and asymptomatic CNS involved patients, atezolizumab and nivolumab seems to be good choices. In OAK trials, atezolizumab provided nearly double median OS than docetaxel for CNS involved patients (20.1 m vs 11.9 m, HR 0.54; 0.31–0.94 95% CI) and longer median time to develop new CNS diseases (not reach vs 9.5 m) [12]. In ASCO 2016, a sum-analysis of checkmate 017/057/063 revealed that nivolumab can prolong survival time (8.4 m vs 6.2 m) with less irAEs comparing to docetaxel [50], and the similar results were achieved by EAP program with more cases in Italy [51]. Keynote 024 is a trial of first line treatment including CNS metastasis patients [20]. 18 patients with CNS metastasis harboring high PD-L1 (TPS ≥ 50%) in pembrolizumab group have better PFS and OS data than the other 10 patients in chemotherapy. In a perspective phase 2 study for pembrolizumab including 18 stable brain metastasis patients with PD-L1 positive (TPS ≥ 1%), the response rate among them is 33% [52], indicating the pembrolizumab is work for selected CNS related patients. Accordingly, though lacking enough perspective evidence, ICIs as monotherapy in advanced NSCLC with brain metastasis has been proved preliminarily.

Radiotherapy is standard local treatment for brain metastatic lesions. To our knowledge, radiation induced inflammatory can promote necrosis of tumor and tumor-associated antigen presented, and further activates T-cell in anticancer immunity [53]. Thence, combination of immunotherapy and radiotherapy may play a synergistic role in advanced NSCLC therapy, which were improved by a secondary analysis of keynote 001 [54]. Herein, the question is whether the adding ICIs would help to add more benefits for CNS metastatic patients. In Pacific study, less new brain diseases were found in arm durvalumab (5.5% vs 11.0%) [55], suggesting that combination of extracranial radiotherapy and ICIs would help to control brain metastasis. Chen et al. reported that patients with brain metastasis in melanoma, nephroma and NSCLC can have significant survival benefits from concurrent immunoradiotherapy comparing to radiotherapy alone or asynchronous immunoradiotherapy (median OS: 24.7 m vs 12.9 m vs 14.5 m) [56]. In a retrospective research including 17 CNS related NSCLC patients, 48% 6-month intracranial ORR was achieved [57]. However, efficacy is varied according to the order of radiation and ICIs. 57% 6-month intracranial ORR was seen in patients who received radiation before or during immunotherapy while 0% in after, indicating processing radiotherapy before or during immunotherapy has better efficacy. Beside efficacy, safety is another important area needs to be explored in depth. Hubbeling HG et al. found that treatment with ICIs and brain radiation wouldn’t improve incidence of radiation related adverse effects (AEs) significantly and no difference in AEs rate base on the order of treatment [58]. However, another retrospective analysis reported that taking ICIs as monotherapy after gamma knife will increase the risk of radionecrosis of intracranial lesion to 37.5% [59]. Thence, combination of immunotherapy and radiotherapy should be carefully selected. Herein, we are looking forward the perspective trails (NCT02978404, NCT02858869), which are conducting for exploring more evidences in the combination of immunotherapy and radiotherapy, to answer the questions in fractionation schedule, radiation dose, target volume and patient selection. More combination strategies for ICIs and other therapies in advanced NSCLC with CNS metastasis are still under exploring. A sub-analysis of keynote 189 with 109 brain metastasis patients, combination of pembrolizumab and chemotherapy significantly extents OS comparing to monochemotherapy. NCT0296993 is a trail for exploring double ICIs plus radiotherapy and NCT02681549 is for combination of immunotherapy and bevacizumab (Table 5).

**Table 5:** Ongoing trials for evaluating combination strategies of checkpoints inhibitor in advanced NSCLC with brain metastasis

Identifier	Title	Phase	Population	Arms	Primary endpoint	Secondary endpoint	Status	Primary completion
NCT02978404	Combining radiosurgery and nivolumab in the treatment of brain metastases	II	Stage IV NSCLC or ccRCC with brain metastasis	Nivolumab (240 mg IV q2 week or 480 mg IV q4 week) and Radiosurgery [15–20 Gray (Gy) in 1 fraction]	Intracranial PFS	Treated brain lesions control rate, OS, PFS, neurocognitive function, toxicity and etc	Recruiting	1-Jun-21
NCT02858869	Pembrolizumab and stereotactic radiosurgery for melanoma or non-small cell lung cancer brain metastases	I	NSCLC or melanoma with brain metastasis	Arm A (pembrolizumab, SRS 6 Gy)	Proportion of dose limiting toxicities	ORR, OS, rate of local recurrence and etc	Recruiting	1-Oct-20
				Arm B (pembrolizumab, SRS 9 Gy)				
				Arm C (pembrolizumab, SRS 18–21 Gy)				
NCT02696993	Nivolumab and radiation therapy with or without ipilimumab in treating patients with brain metastases from non-small cell lung cancer	I/II	Stage IV NSCLC with brain metastasis	Arm A (nivolumab, SRS)	RP2D and Intracranial PFS	Neurocognitive changes	Recruiting	31-Dec-20
				Arm B (nivolumab, WBRT)				
				Arm C (nivolumab, ipilimumab, SRS)				
				Arm D (nivolumab, ipilimumab, WBRT)				
NCT02681549	Pembrolizumab plus bevacizumab for treatment of brain metastases in metastatic melanoma or non-small cell lung cancer	II	NSCLC or melanoma with brain metastasis	Pembrolizumab plus bevacizumab	BMRR	ORR, PFS, safety and toxicity, biomarkers for efficacy prediction	Recruiting	1-May-21

Systematic immunotherapy has been an important part for advanced NSCLC, but the efficacy in CNSs metastasis patients is still under exploring

*RP2D* recommended phase 2 dose, *BMRR*, brain metastasis response rate, *SRS* stereotactic radiosurgery, *WBRT* whole-brain radiotherapy

## Neoadjuvant immune checkpoint inhibitors in NSCLC

Surgery is the only radical treatment strategy for resectable NSCLC currently [60]. However, 5-year survival rate after surgery is low, vary from 19 to 73% [61]. Theoretically, neoadjuvant therapy can eliminate micro-metastatic diseases before surgery to reduce the rate of recurrence. However, preoperative chemotherapy could only increase 5% survival benefits [62]. As for perioperative radiotherapy, it helps to improve local control rate, but seems to contribute nothing to survival benefits [63]. Immune checkpoint inhibitors have the opportunity break the bottleneck of current neoadjuvant therapy. Forde et al. firstly reported a pilot study of nivolumab as neoadjuvant therapy for stage II–IIIA NSCLC [8]. After taking 2 cycles nivolumab, 20/21 patients received operation, 45% (9/20 cases) major pathological response (MPR) was achieved with acceptable side-effect profile. Moreover, 15/20 patients are disease-free and alive for 30 months [64]. Tumor specific T-cell clones were observed in both resected samples and peripheral blood samples, indicating that ICIs can activate T cells systematically to eliminate both primary and metastatic tumors including micro-metastasis, which was more significant in MPR patients. Comparing to the expression of PD-L1, TMB is better for MPR prediction. Recently, an extended analysis of this study indicates that ctDNA and peripheral T cell expansion are potential biomarkers for response and surveillance prediction [64]. Interestingly, there is great disunity between pathologic and radiologic diagnosis in the evaluation of immune neoadjuvant therapy efficacy (RECIST: 2/20 PR, 18/20 SD; Pathology: 9/20 MPR), suggesting that current RECIST standard is not suitable for evaluation. The percentage change of standard uptake value (SUV) in positron-emission tomography scan (PET/CT) could be a better evaluation standard for neoadjuvant immunotherapy since it reflects the metabolic activity of cell, which is corresponded to pathological response [65]. According to the interim analysis of LCM3, atezolizumab is effective and safe as neoadjuvant therapy for stageIB to selected IIIB NSCLC patients [66]. Among 82 resected patients, 15 cases (18%) achieved MPR with only 6% grade 3/4 treatment related adverse effects occurred. Interestingly, biomarkers for MPR prediction are quite different to Forde PM’s study. MPR rate is not significantly different between PD-L1 negative and positive (cutoff: 1%; clone sp142, 2/26 vs 10/35, p = 0.055). However, it is meaningful in low and high expression (cutoff: TPS = 50%; clone 22c3, 5/44 vs 7/20, p = 0.040). Moreover, median TMB (10.4 Mut/Mb) is not different in patients with MPR or not. Thus, patient selection for neoadjuvant immunotherapy is still controversial. Several ongoing trials are further exploring the dose, administration time, safety and efficacy of neoadjuvant ICIs for early stage NSCLC (LCM3 for atezolizumab, NEOSTAR for nivolumab, MK3475-223 for pembrolizumab).

Given the excellent synergistic effects of the combination of immunotherapy and other therapies in advanced NSCLC, several trials have been designed to evaluate the feasibility and safety of combination strategies in neoadjuvant therapy. Results of NADIM (NCT 03081689) presented in ASCO 2019 demonstrated that 41/46 stage IIIA patients were performed R0 resection after 3 cycles neoadjuvant immunochemotherapy (nivolumab + paclitaxel + carboplatin), 34 patients (83%) achieved MPR including 24 (71%) of them were complete pathologic response (pCR). Moreover, 90% patients experienced downstaging [67]. MK-3475-671 is a double blind phase III study in comparing the efficacy of perioperative pembrolizumab or placebo plus platinum based neoadjuvant chemotherapy for stageIIB orIIIA NSCLC, which will answer the question whether patients can earn more benefits from adding ICIs in neoadjuvant chemotherapy. As we know from keynote-001, prior radiation would prolong PFS for patients received ICIs. Ongoing pilot trials NCT 03237377 is exploring the safety of neoadjuvant immunoradiotherapy for resectable IIIA NSCLC (durvalumab + radiation or durvalumab + termelimumab + radiation). Synergistic effect of ICIs-based combination in neoadjuvant therapy is another active research area. Data from NEOSTAR (ASCO 2019), higher median percentage of non-viable tumor (viable tumor: 20% vs 65%, p = 0.095) and of tissue residual memory TILs (CD3+:81.2% vs 54.4%, p = 0.028; CD8+: 56.2% vs 38.3%, p = 0.069) were seen in nivolumab plus ipilimumab group comparing to nivolumab alone [68]. Further analysis is still ongoing. Though efficacy and safety of combination strategies were preliminarily proved, patient selection is still unclear. Therefore, more studies are required to explore better biomarkers for patient selection of combination neoadjuvant therapy.

## Potential immune checkpoint inhibitors

Though immune checkpoint inhibitors targeting in PD-1/L1 axis have achieved great progression in the treatment of NSCLC, the proportion of beneficiaries is still low currently [17]. Recent researches in other immune checkpoints are expected to expand the population benefiting from immunotherapy. NKG2A is an intracytoplasmic tyrosine-based inhibitor motif, expressed in NK cells and selectively in CD8+ CTLs in tumor microenvironment, which can block the immune ability of NK cell and CD8+ T cell if it binds its ligand (HLA-E) overexpressed in carcinoma of lung, cervix and head/neck [69]. Interestingly, anti-NKG2A antibodies can play anti-cancer role only in inflammatory tumor environment [17], indicating they need to be combined with other agents to kill tumor. Monalizumab is a humanized anti-NKG2A antibody, which can enhance the anti-tumor ability of NK cells, and can rebuild the anti-tumor ability of CD8+ T cells when blocked with PD-1/PD-L1 axis at the same time. The preclinical study demonstrated that durvalumab alone can save about 40% mice from death, while combining with monalizumab can achieve an efficacy of 75% [17]. In addition, its efficacy and safety were evaluated in a phase II clinical trial of monalizumab and cetuximab in the treatment of recurrent squamous cell carcinoma of the head and neck. The mid-term analysis showed that the effect of dual drug combination was better than single drug of cetuximab, with an ORR of 31% [69]. As for NSCLC, one arm of PIONeeR-Clinical study is exploring the efficacy of combination of durvalumab and monalizumab in advanced NSCLC resistance to ICIs. And the efficacy in neoadjuvant therapy for NSCLC is evaluating in NeoCOAST. Ecto-5′-nucleotidase (CD73) is an enzyme highly expressed in anergic T cell, which degenerates AMP to adenosine leading to inhibit CTLs function in anticancer immunity. Additionally, CD73 can induce angiogenesis and lymphangiogenic to promote the development and progression of tumor [16]. Therefore, antibody targeting in CD73 would inhibit the growth of cancer through reducing the production of adenosine to relieve its inhibitory function in CTLs and blocking angiogenesis and lymphangiogenic. Indeed, preclinical studies have demonstrated the anti-cancer abilities of anti-CD73 antibodies in several kinds of cancer. MEDI9447 (oleclumab) is a humanized anti-CD73 antibody [70]. Given the anti-cancer ability of oleclumab alone or in combination with durvalumab in vivo and animal model, a phaseIstudy (NCT02503774) is conducting for evaluating the safety, tolerability, pharmacokinetics, immunogenicity, and antitumor activity of MEDI9447 alone and in combination with durvalumab in adult subjects with select advanced solid tumors. However, since CD73 is involved in homeostasis regulation such as epithelial barrier function and intestinal secretion and reabsorption function, attention should be paid to the possible adverse effects when using CD73 [16].

Adaptive resistance to PD-1/L1 inhibitors is hampering the further progress of immunotherapy [14]. Koyama et al. reported that the selective activation of T-cell immunoglobulin mucin-3 (TIM3) is the key mechanism of resistance of anti-PD-1 immunotherapy [15]. Mice models (lung cancer) revealed that CD8+ T cells would fail after anti-PD-1 resistance, which was related to the up-regulation of multiple immune checkpoints expression, especially TIM3. Furthermore, mice can earn more survival time from sequential blocking therapy with TIM3 after anti-PD-1 treatment than anti-PD-1 alone (11.9 weeks vs 5 weeks, p = 0.0008). Additionally, high levels of TIM3, rather than other immune markers, were detected in T cells of NSCLC patients who had been treated with anti-PD-1 and developed drug resistance. Therefore, inhibiting TIM3 would help to overcome the adaptive resistance to anti-PD-1. An interim analysis of AMBER study, posted in SITC 2018 meeting [71], revealed that for patients with advanced NSCLC after resistance to PD-1/L1 antibody, 9% ORR was achieved by 100 mg TSR-022 (anti-TIM3) combined with 500 mg TSR-042 (anti-PD-1) and 15% by each 300 mg, with acceptable adverse effects (6.7% grade ≥ 3). It is worth mentioning that for those expressed more than 1% of PD-L1 in both groups, total ORR reached 33%.

**Fig. 1:**
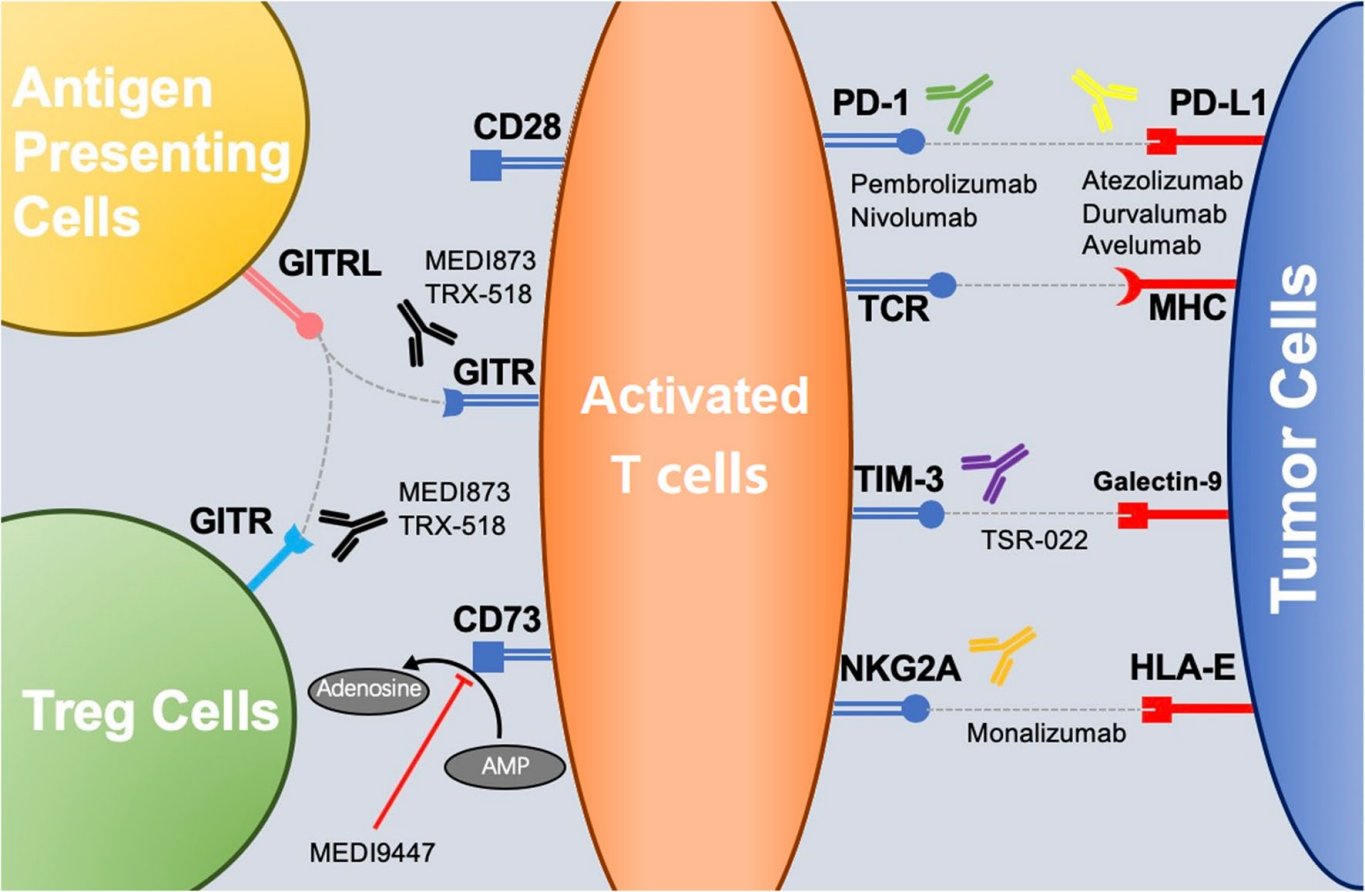
Immune checkpoints with potential therapeutic value in NSCLC. Reported immune checkpoints inhibitors with therapeutic value in NSCLC are illustrated. Because of the limited beneficiary population and resistance to ICIs against PD1/L1 axis, novel agents targeting in other immune checkpoints are developed to overcome the currently limitation of ICIs. After the recognition of TCR and MHC, costimulatory factors help to regulate the activation of T cells. The stimulation of positive checkpoints and inhibition of negative checkpoints are to maintain or recover the anticancer ability of T cells. Antibodies targeting negative checkpoints (PD-1/PD-L1, TIM-3/Galectin, NKG2A/HLA-E, CD73) are to release the inhibition of T cells, while agonists of positive checkpoints (GITR/GITRL) are to strengthen the activation of T cells

Great proportion of patients treated with single agents PD-1/L1 inhibitors could not develop durable anticancer response because of the inability to produce long-term immunological T cell memory. Current studies on costimulatory pathway demonstrated great potential to improve efficacy of checkpoint inhibition and induce durable anticancer response. GITR [glucocorticoid-induced tumor necrosis factor receptor (TNFR)–related protein] belongs to TNFR super family, which can activate anticancer ability of CD4+ and CD8+ T cells and block the inhibitor effect of Treg on CTLs [72]. Previous clinical studies revealed limited efficacy in monotherapy with agonists of GITF, but great potential value in combination of agonist of GITR and anti-PDl/L1. MEDI1873 (GITR agonist) was proved to activate CD4+ T cell in peripheral blood and eliminate Treg intratumor (NCT02583165), while TRX518 (GITR agonist) is able to clear Treg cells but unable to reverse the deletion of CD8+ T cells without combination with PD-1 antibody (NCT02628574). Wang et al. found that activating GITR combined with blocking PD1 can effectively reverse the depletion of CD8+ T cells and maintain the phenotype of memory T cells. The clearance of Treg by GITR antibody provided immune activation relief to CD8+ T cells, and the proliferation of CD8+ T cells intratumor was significant [73]. Results from ASCO 2018 meeting, revealed that under the condition of 170 mg alone and 60 mg in combination, MK-1248 (GITR agonist) had good tolerance, no dose-limiting toxicity and no treatment-related death for advanced solid cancer [74]. AS soon as combined with pembrolizumab, the therapeutic response was observed (1CR, 2PR; NCT02553499) (Fig. 1).

## Conclusion

The rapid progression of immunotherapy in recent years has broken through the bottleneck of cytotoxicity-based chemotherapy in wild-type non-small cell lung cancer and improved the prognosis and life quality for patients. However, due to the little effective population of single drug treatment, the clinical application of ICIs is greatly limited. Synergistic effects in combination based on ICIs are expanding the beneficiary population. To be noticed, there are still lots of problems being eager to answers, such as the patient selection for best combination strategy, the identification of pseudoprogression, administration sequencing and so on. Moreover, combinations of PD-1/L1 blockage and novel immune checkpoint inhibitor targeting other than PD-1/L1 axis are preliminary proved to be worth looking forward to overcome primary or adaptive resistance of anti-PD-1/L1 antibodies. Predictably, more novel agents and combination strategies will help to NSCLC treatment. However, how to achieve precise immunotherapy for maximizing the benefits of patients is worthy of our in-depth exploration in the future.

## Data Availability

Not applicable.

## Abbreviations

CTL: cytotoxic T lymphocyte
TPS: tumor proportion score
ALK: anaplastic lymphoma kinase
CNS: central nerve system
CPI: checkpoint inhibitor
DCR: disease control rate
EGFR: epidermal growth factor receptor
IgG1: immunoglobulin G1
NSCLC: non-small cell lung cancer
ORR: objective response rate
OS: overall survival
PD-1/PD-L1: programmed cell death protein-1/programmed cell death protein ligand-1
PFS: progression-free survival
TKI: tyrosine kinase inhibitor MPR: major pathological response
TMB: tumor mutation burden
